# Silent witnesses: unveiling the epidemic of femicides in North-west Tshwane, South Africa – a decade of analysis

**DOI:** 10.1186/s12889-024-21059-7

**Published:** 2025-01-29

**Authors:** Yaseen Bismilla, K. K. Hlaise, C. Van Wyk

**Affiliations:** 1 Sefako Makgatho University, Ground Floor, Clin Path Building, Room No. 32. Garankuwa, Pretoria, South Africa; 2 Sefako Makgatho University, Ground Floor, Clin Path Building, Room No. 39. Garankuwa, Pretoria, South Africa; 3 Sefako Makgatho University, Ground Floor, Clin Path Building, Room No. 37. Garankuwa, Pretoria, South Africa

**Keywords:** Gender based violence, Femicides, Medico-legal mortuary, Forensic medicine, Community-based violence prevention, Epidemiology, Demographics, Pathology

## Abstract

**Background:**

Femicides, defined as the gender-based killing of women, are a pressing public health issue worldwide, with South Africa experiencing some of the highest rates globally. This study focuses on the North-west region of Tshwane, particularly the Garankuwa area, aiming to address gaps in understanding the epidemiology, demographics, circumstances, and pathology associated with femicides. The Garankuwa mortuary serves as the primary site for this investigation, providing a detailed analysis over a ten-year period, shedding light on contributing risk factors in the context of systemic gender inequality.

**Objectives:**

The study had four main objectives: to analyse the demographics and incidence rates of femicides in the North-west Tshwane area, to examine the circumstances surrounding femicides (including the time, location, and demographic risk factors), to identify the causes and pathological characteristics of femicides; and to observe trends in femicide rates over the ten-year study period.

**Methods:**

This study was a retrospective cross-sectional descriptive analysis, focusing on all deceased females admitted to the Garankuwa mortuary from 2009 to 2018. The inclusion criteria comprised female cases at autopsy, with a suspected homicidal manner of death. Excluded were cases classified as suicides, accidents, or natural deaths after further examination. Data was collected through the National Injury Mortality Surveillance System (NIMSS) and cross-verified with post-mortem reports, police data and death registers.

**Results:**

Over the ten-year period, the Garankuwa mortuary admitted an annual average of 1131 bodies, with approximately 23.5% (266) being female. Of these, 17.5% were identified as femicides. The average incidence rate of femicides was 11.2 per 100,000 female population, showing a general decline over the study period, except for notable increases in 2013 and 2016. The study found that the most common months for femicides were September and December, with most incidents occurring at home, followed by residential areas and medical facilities. The geographic analysis identified Temba, Rietgat, and Akasia as the areas with the highest number of femicide cases. Demographically, the majority of femicide victims were black, with the most affected age group being 18–39 years. The leading causes of death were gunshot wounds, sharp force injuries and blunt force trauma, with significant incidences of strangulation and asphyxial deaths, primarily affecting the neck and head regions.

**Conclusion:**

This study highlights the high incidence and distinct characteristics of femicides in the North-west Tshwane area, underscoring the role of socio-economic disparities and racial demographics. The findings emphasize the necessity for targeted prevention programs, stricter firearm control measures, and community-based violence prevention strategies. The demographic data indicate that young black women are particularly vulnerable, necessitating protective and educational initiatives tailored to this group.

## Introduction

Gender-based violence (GBV) refers to harmful acts directed at an individual or group of individuals based on their gender, and is primarily rooted in gender inequality, the abuse of power and harmful norms [1, 2]. GBV is an increasingly serious South African public health concern and international human rights issue [3].

Violence against women (VAW) is defined as any act of gender-based violence that and encompasses physical, sexual, psychological, emotional and financial abuse to women. VAW specifically is the most common form of GBV [1]. It is reported that worldwide, at least one in five women have been physically and/or sexually abused by a man or men at some point of their lifetime [3].

Violence against women and adult femicides are critical medico-legal issues nationally and worldwide. The South African and international media constantly report on the murder of women. Year 2013 and 2014 in particular were populated by global media headlines involving the killing of two South African women namely Annene Booysen and model Reeva Steenkamp [4].

In Booysen’s case, who was brutally raped and murdered in a marginalized community, socio-economic deprivation and limited access to resources reflect a context in which gender-based violence is widespread and often normalized. In economically disadvantaged communities, factors like poverty, inadequate education, and systemic neglect create environments where patriarchal norms can thrive unchallenged. In contrast, Steenkamp’s death, while occurring in a more affluent context, reveals that South Africa’s deeply embedded gender inequalities transcend economic class. Her killing highlights how entrenched gender biases and societal attitudes toward power dynamics can lead to lethal outcomes. Both cases reflect South Africa’s broader context, where socio-economic disparities and gender inequality intersect to fuel a persistent culture of violence against women [5].

“Femicide” refers to the intentional killings of women and girls, representing the lethal end point of multiple, overlapping, and interconnected forms of gender-based violence. It is characterized by its unique motivations, which stem from systemic gender inequality, misogyny, and discriminatory social norms. Femicide encompasses a range of acts where the victim’s gender is the key motivating factor, often as part of domestic and sexual violence. This differentiates it from other homicides involving female victims where gender is not the primary motive, such as deaths that result from robberies, gang violence, or other non-gendered criminal activities. These latter types of female homicides are typically incidental to broader criminal activities and are not motivated by the victim’s gender. The specificity of femicide lies in its basis in gender discrimination, which directly influences both the perpetrator’s intent and societal responses to such violence [4].

South Africa has one of the highest femicide rates in the world. High incidence rates of femicide have been recorded by the South African Police Service (SAPS) in their annual crime reports, with 989 women murdered during the 2021 to 2022 financial year, an alarming 14.1% increase from the previous financial year [6].

Femicide contravenes women’s constitutional right to life as outlined in the International and Supreme laws of most countries, including South Africa [7]. The first National femicide study in South Africa was undertaken in 1999 and reported an incidence rate of 24.7/100,000 for women 14 years and older. This incidence rate is six times higher than the international rate of 4.0/100,000 population as estimated by the World Health Organization at the same time [3].

Regarding the incidence of femicides, global figures differ from one country to another. The United States in 2011, reported a femicide incidence rate of 4.7 / 100,000 women, with a 22.4% femicide rate [8]. In Taiwan, Wen-Li Fong et al. noted femicide rates to be slightly higher, accounting for 29.1% of all homicides in 2010 [9].

Despite the magnitude of deaths resulting from VAW nationally and globally, there are still gaps in data that undermine prevention efforts [10]. There is limited research regarding femicides in the specific location of this study, providing such gaps in data.

The Garankuwa mortuary functions as a teaching facility for the Sefako Makgatho Health Sciences University. The mortuary handles an estimated 1200 autopsy cases per year. The North-western part of the City of Tshwane, which includes the areas of Akasia, Rosslyn, Pretoria North, Klipfontein, Ga-Rankuwa, Mabopane, Winterveld, and Soshanguve, as well as a rural zone in the west, has a population of approximately 997,000, making it the most populous region in the City of Tshwane [11].

Historically, Ga-Rankuwa faced substantial socio-economic challenges due to its establishment during apartheid, when townships were created as racially segregated areas. It remains a mix of urban and larger rural areas, with lower-income and working-class households, facing limited access to economic opportunities and social services [12].

This study will help identify vulnerable women in the North-western District of Tshwane and assist in developing tools to implement a targeted, context-specific femicide prevention programs to protect women from the hazards of violence that threaten their lives in this area. Moreover, this study emphasizes the need for raising awareness about the scale of female violence in society.

## Methods

This study is a retrospective, cross-sectional descriptive analysis focusing on all deceased females admitted to the Garankuwa mortuary from January 1, 2009, to December 31, 2018, where the apparent manner of death was suspected to be homicidal.

The inclusion criteria consisted of cases involving females or individuals of undetermined sex confirmed as female via autopsy or anthropological analysis in cases of severe decomposition, with a suspected initial manner of death as homicide or unknown. Exclusion criteria included cases identified as suicides, accidents, or natural deaths upon further investigation; cases lacking supporting evidence for homicide and awaiting toxicology or histology results; decomposed or skeletonized cases without supporting historical or forensic findings; non-viable abandoned fetuses under 26 weeks; and cases with post-mortem reports pending finalization.

The study used pre-validated data collection tools, improving the validity of the results. Data was extracted from the department’s electronic database using the National Injury Mortality Surveillance System (NIMSS) data collection form, which is attached to every post-mortem case. This electronic data base includes information such as gender, race, age, SAPS location, scene of injury, date and time of death, and apparent manner of death, assisting in the initial sorting by inclusion criteria. Reliability was enhanced by cross-referencing NIMSS data with post-mortem reports, docket identification forms, and death registers to complete any missing information. The researcher double-checked the data collection and capture, ensuring further reliability.

Limitations for the study include cognitive bias by individual pathologists, since the cases have been concluded and classified as homicide, the researcher did not review or re-classify individual cases. Cognitive bias by individual pathologists, as noted above, is identified and declared, as this may have influenced the interpretation of post mortem findings and classification of cases.

Docket information for each case at the Garankuwa mortuary is securely stored in an access-controlled, locked storeroom. The annual death registers, post-mortem reports, NIMSS form, autopsy scribe notes, supporting information regarding the deceased’s history and identity, and results from ancillary investigations were manually reviewed by the researcher to determine the cause of death, affected body regions, and the number of inflicted wounds.

Data capture involved extracting information from the database, the death register, and post-mortem reports. The researcher used a data collection sheet to capture this information electronically in Microsoft Excel (Microsoft Office 365) and IBM SPSS statistical software for subsequent analysis.

## Results

From the totals obtained for North-western Pretoria female population were estimated by multiplying Tshwane population by the average percentage of Region 1 (27%) and then by the 0.5 to estimate female population (Table 1). It shows that there was an estimated annual average population of 419,273 females in the in the North-west area of Tshwane serviced by Garankuwa mortuary. This number represents about 50.5% of the population of the entire North-western Tshwane region during the study period [11].

During the ten-year study period, an annual average of 1131 bodies were admitted at the Garankuwa mortuary, of which an overall average of 266 bodies were female. Of all the female bodies admitted to Garankuwa mortuary over this period, an average of 17.5% (46.5 bodies) were noted to be femicides (Table 1).

The average incidence of femicides in the North-western region of Tshwane was 11.23/100,000 female population over the study period. The incident rate generally declined over the 10-year study period, with exceptions in 2013 (13.0/100,000) and 2016 (10.9/100,000), which showed increases. The highest incidence was observed in 2009 (16.1/100,000) and the lowest in 2012 (8.6/100,000).

**Table 1:** Number and incidence rates of femicides per population of North-west Pretoria

Year of study	Total no of bodies admitted to Garankuwa FPS	Total female bodies admitted to Garankuwa FPS	Total estimate female population of North-west Pretoria (Region 1)	Total femicides in North-west Pretoria	Femicide incidence per 100,000 population in North-west Pretoria
2009	1186	259	366,338	59	16.1
2010	2090	263	378,206	56	14.8
2011	1026	255	390,674	53	13.6
2012	1016	259	402,977	35	8.6
2013	1045	272	414,734	54	13.0
2014	1157	274	426,139	36	8.4
2015	1167	268	437,201	40	9.1
2016	1237	283	448,027	49	10.9
2017	1172	266	458,885	41	8.9
2018	1219	264	469,558	42	8.9
**Average**	**1131**	**266**	**419**,**273**	**46.5**	**11.23**

**Fig. 1:**
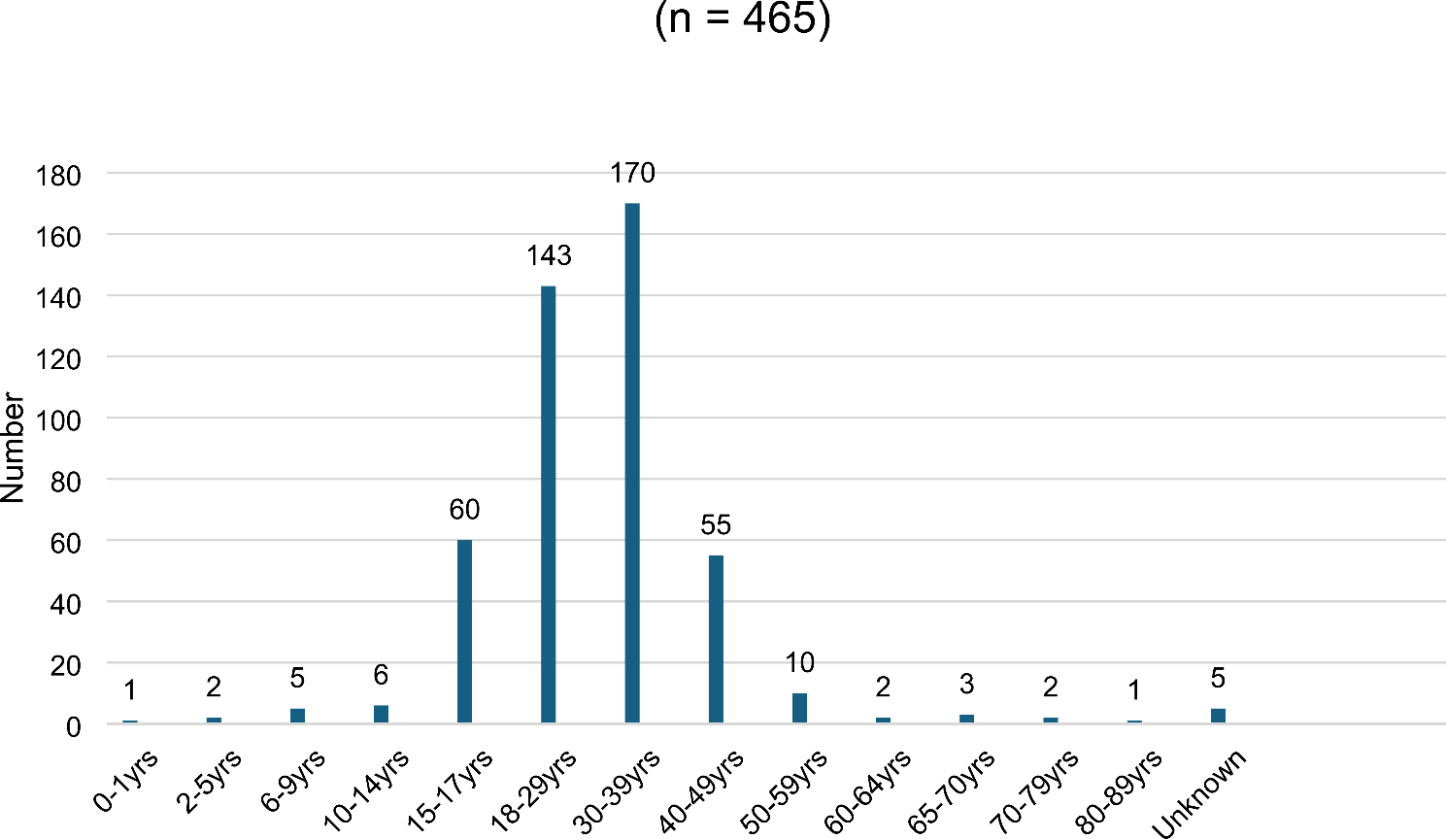
Distribution of femicides by age

The youngest victim was a newborn (1 case) and the oldest was 88 years of age (with 5 cases having undocumented ages) (Fig. 1). The median age was 44. The highest age groups of femicides in this study were in the age group 30–39 years old, 36.6% (*n* = 170), closely followed by group 18–29 (*n* = 143, 30.8%), the 15–17 years (*n* = 60, 12.9%) and 40–49 (*n* = 55, 11.8%). These 4 age groups accounted for 92% of all femicides during the study period. The child femicide age group (0-17-year-old) accounted for 15.9% of all femicides (*n* = 74), adults (18-64-year-old) accounted for 81.7% of all femicides (*n* = 378) and the elderly (over 65 years old) accounted for 2.4% of all femicides (*n* = 13). The highest age group (18–39 years old) accounted for 67.3% of all femicides.

With regards to race distribution, the majority of cases in this study were black at 97.2%, followed by white at 2% and other races at 0.8%. The most common month of Femicide occurrences was December (3 of the 10 years) and September (2 of the 10 years), with January (3 of the 10 years) and July (3 of the 10 years) equally being the least common month. The areas where the bodies were mostly recovered showed to be highest in the home (40.2% of all cases), followed by residential area (19.2% of all cases), then from a medical facility (7% of all cases).

**Fig. 2:**
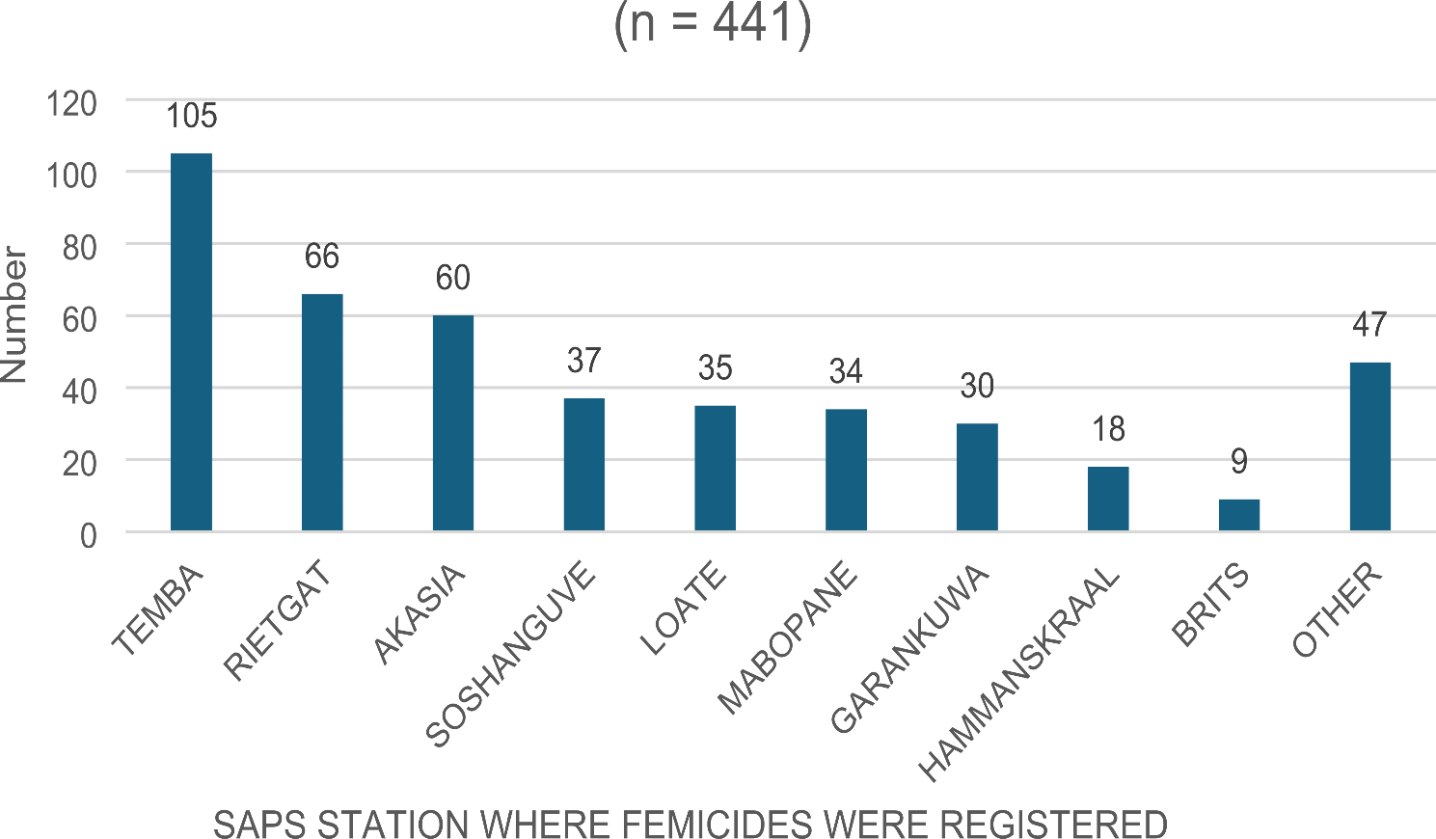
Distribution of femicides by SAPS location

25 cases did not have a SAPS station, giving the total of 441 cases analyzed (Fig. 2). The top three SAPS stations where the most femicides occurred were Temba 24.0% (*n* = 105), followed by Rietgat 15.0% (*n* = 66), lastly Akasia 13.6% (*n* = 60). Therefore, Temba SAPS accounted for the highest femicides during the study period. The “other” category showed a high number of cases, as over 30 SAPS stations were represented in this category.

**Fig. 3:**
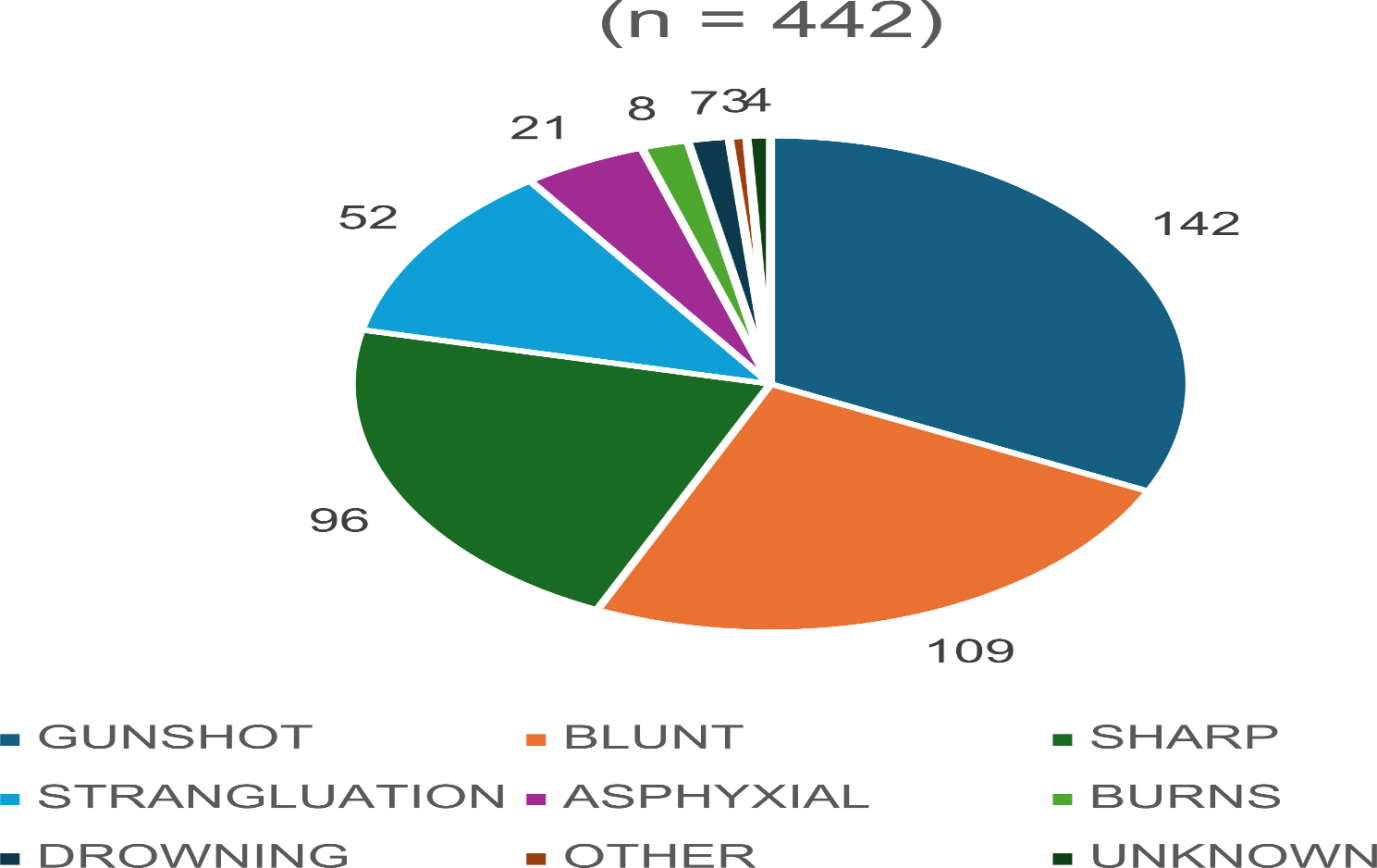
Distribution of femicides by causes of death

Of the total number of cases, 95% (*n* = 442) had confirmed causes of death (Fig. 3). The other 24 cases were missing or undocumented. It can also be seen that most victims died from gunshot injuries, accounting for 32.1%, (*n* = 142) of cases, followed by blunt force injuries (24.7%, *n* = 109) and sharp force trauma (21.7%, *n* = 96). Strangulation deaths accounted for 11.7% (*n* = 52) of cases, followed by asphyxia-related deaths accounting for a total of 4.7% (*n* = 21) of cases. The remaining causes (burns, drowning, other, unknown) accounted for 5.0% (*n* = 22) of cases.

**Fig. 4:**
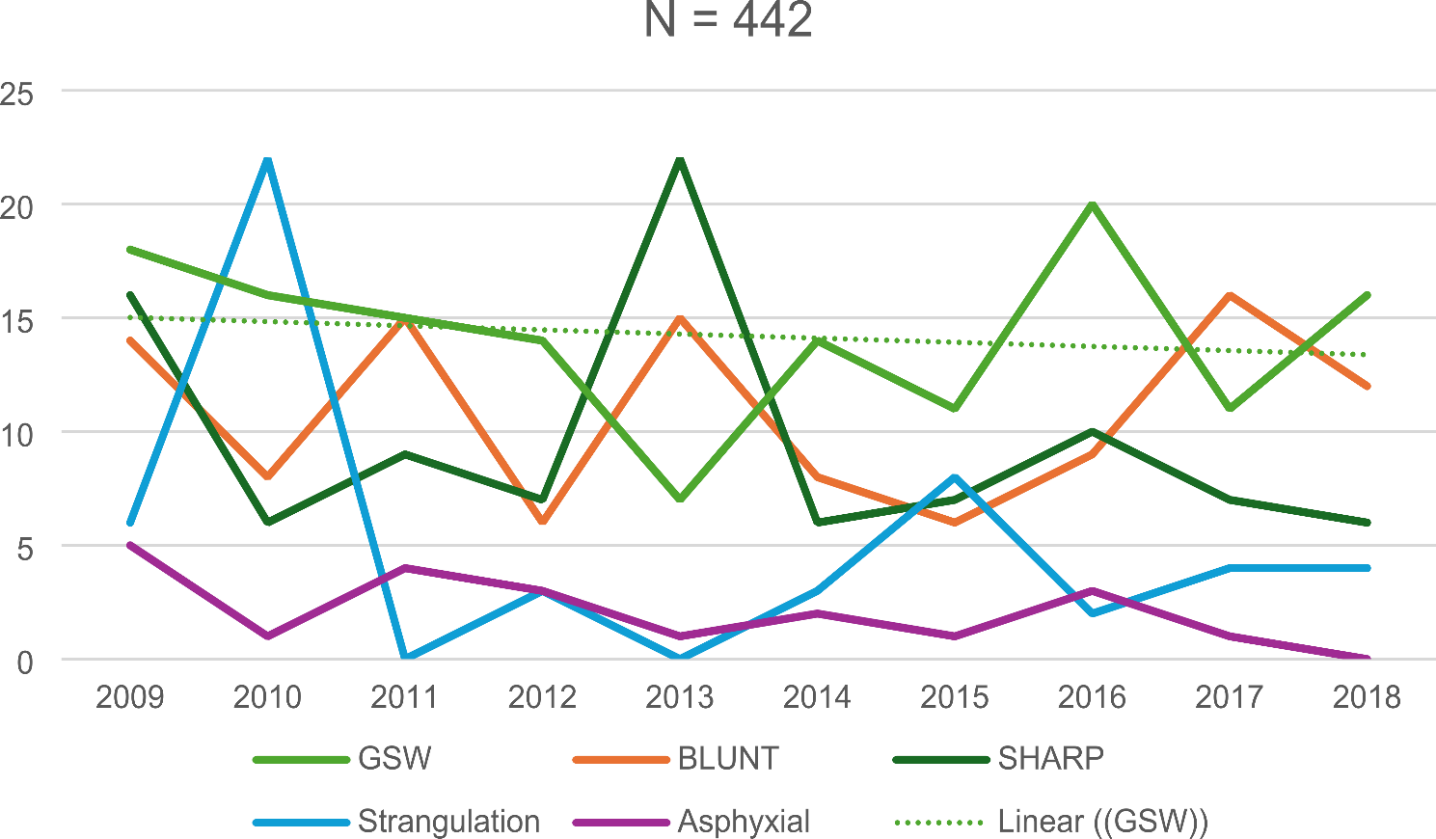
Trends of the top five most common causes of death

Over the 10-year study period, gunshot wounds (GSW) were the most common cause of death, except for 2013 where sharp force injuries were the most common (Fig. 4). Strangulation related killings was one of the least common causes of death throughout the study period, except in 2010, where it was the most common.

**Fig. 5:**
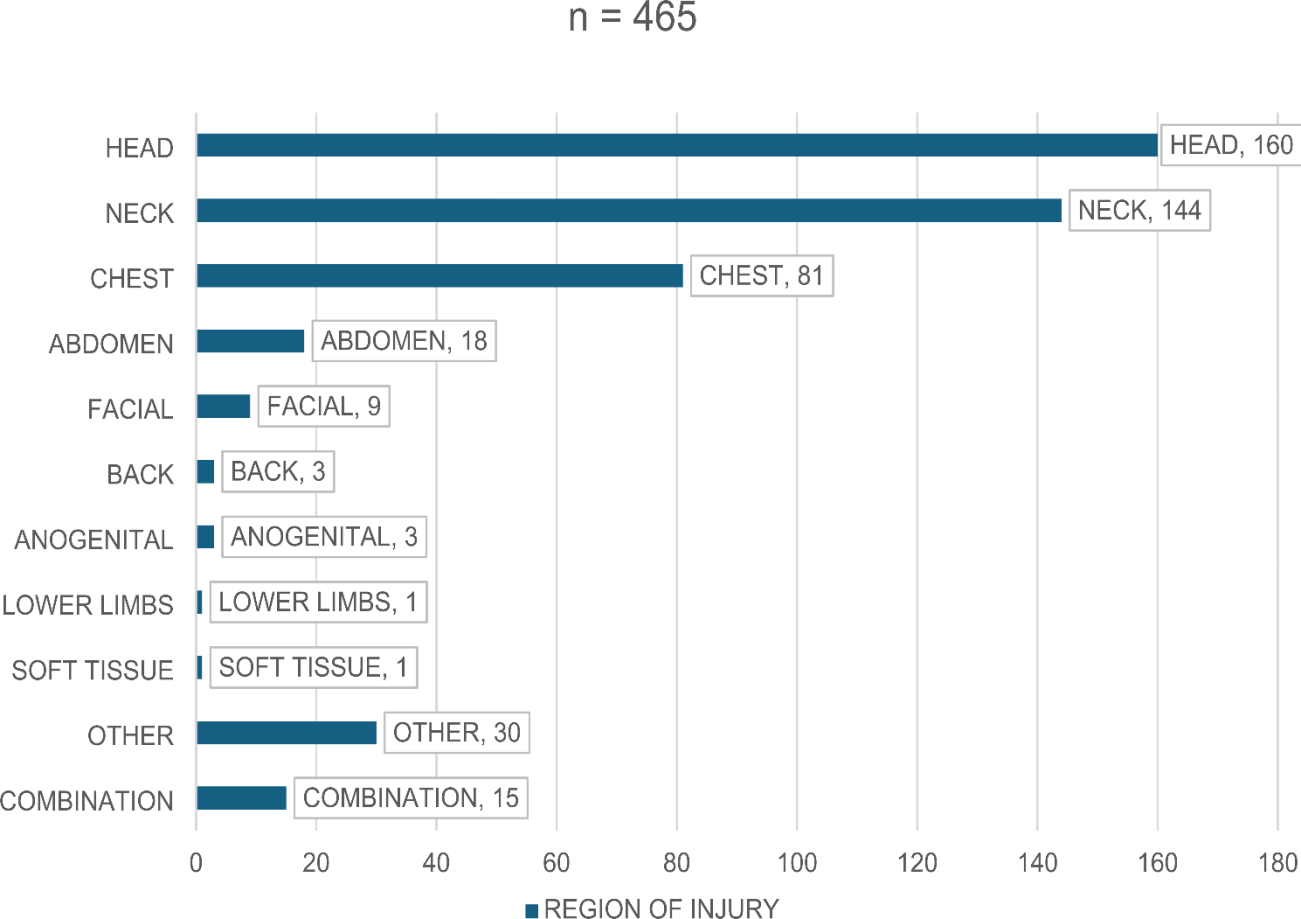
Distribution of femicides by region of injury

The top 3 highest regions of the body involved was the head with 34% (*n* = 160), followed by neck 31% (*n* = 144), and chest at 17% (*n* = 81) (Fig. 5). Facial injuries accounted for 1% (*n* = 9) and anogenital injuries accounted for 0.6% (*n* = 3) of bodies. The remainder of regions involved were unremarkable, however the “other” category accounted for 6% (*n* = 30), which included multiple regions / mass regions involved.

**Fig. 6:**
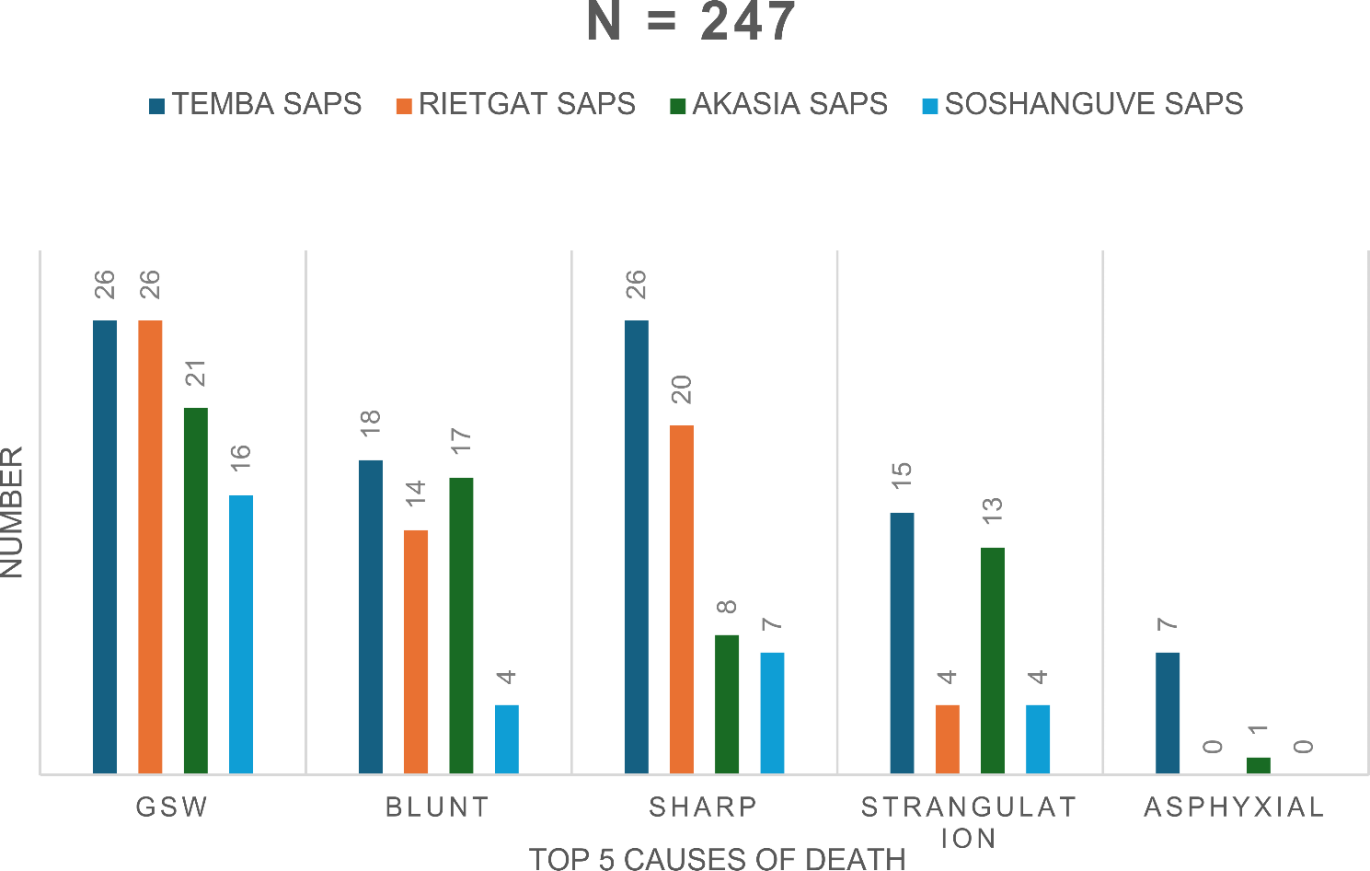
Top five causes of death by SAPS location

The top 4 SAPS locations showing the top 5 causes of death accounted to 247 cases, which represents 56% of all the SAPS stations in this study (Fig. 6). Temba SAPS overall showed the highest number of deaths in all the top 5 causes of deaths. Temba SAPS and Rietgat SAPS showed the highest number of GSW deaths, totaling to a combined value of 56% (*n* = 52) out of the top SAPS locations. After Temba (*n* = 18), Akasia SAPS showed a similarly high number of blunt force femicides (*n* = 17). During this period, Temba SAPS showed the same number of sharp force deaths as GSW (*n* = 26 in both circumstances). Temba (*n* = 15) and Akasia (*n* = 13) also showed the highest rates of strangulation deaths.

**Fig. 7:**
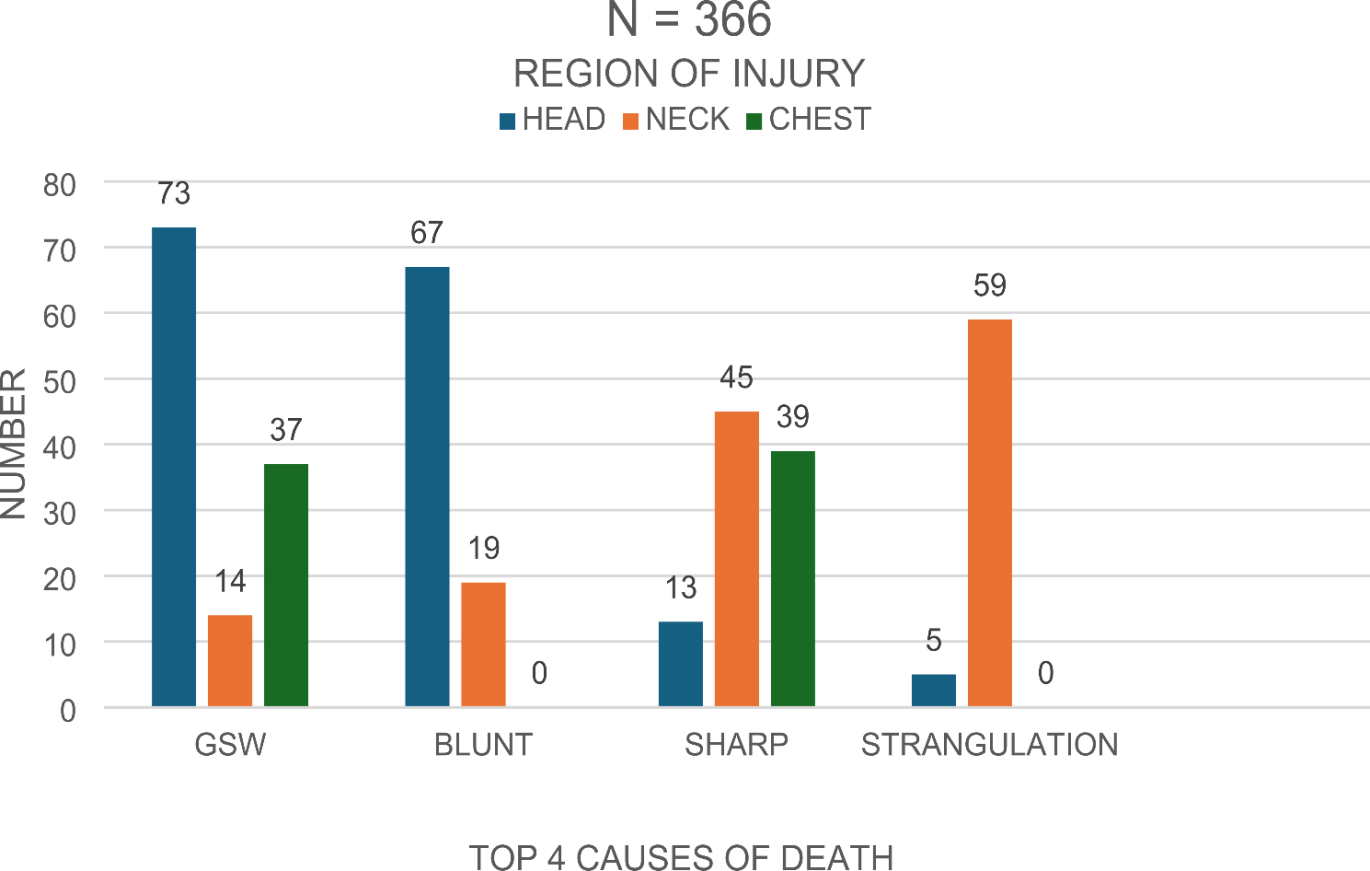
Top three causes of death by region of injury

The highest region of killing to the head was GSW (*n* = 73) accounting for 58% of GSW related deaths to the top three regions (Fig. 7). This was followed by GSW to the chest region with 30% (*n* = 37) of GSW related deaths to the top three regions involved. Blunt force injury was higher in the head region with 78% (*n* = 67) of the top three regions involved in blunt force killings.

Sharp force injury to the neck was the highest region involved, accounting for 46.3% (*n* = 45), closely followed by the chest at 40% (*n* = 39) out of the top three regions involved in sharp force injuries. The neck was the main region of strangulation related deaths in almost all cases, accounting for 92% (*n* = 59) as compared to the other top three regions involved in strangulation deaths.

Of the 442 victims mentioned in Fig. 3, just over half (55%, *n* = 243) victims died from single injuries while 40% (*n* = 177) suffered multiple injuries. The primary medical cause of death was unknown in 5% (*n* = 22) of cases.

## Discussion

In South Africa, specific community issues like women’s isolation, poverty and unemployment play a significant role in increasing the risk of VAW. Isolation limits women’s access to support and resources, leaving them vulnerable to abuse, especially in communities where patriarchal norms restrict their autonomy. Poverty exacerbates this, as financial instability can fuel domestic violence, with men often directing frustration toward women. Unemployment similarly affects women’s health, as it increases their vulnerability to control and abuse, leaving them trapped in harmful environments. Together, these factors underscore the pervasive gender inequalities that systematically disadvantage women, perpetuating cycles of violence and limiting their agency and safety within their communities [13].

This study aimed to explore the epidemiology and pathology of femicides in the North-western region of Pretoria over a ten-year period. The findings have provided significant insights into the demographic characteristics, causes of death, and trends over time, aligning with and expanding upon previous research.

Previous data does not point to specific causes of femicides, but the discussion rather emphasizes the risk factors associated with femicides. Women’s biological factors like age, race and personal history (pregnancy, socio- economic and marital status) all increase the risk of violent fatalities [2]. Information regarding the personal history of femicides in this study was not available at the time of autopsy, as such information is still under investigation.

In South Africa, as in many other countries, reliable national statistics on intimate partner femicides remain scarce [10]. Addressing this gap requires not only robust surveys but also the establishment of better infrastructure for data collection.

This study showed an average incidence rate of 11.23/100 000 in North-west Tshwane area, which is on par with the last reported South African national femicide rate of 11.2/100 000 in 2017 [3]. This average femicide rate of 11.23/100 000 is significantly higher than the United States in 2011, reporting a femicide incidence rate of 4.7 / 100,000 women [8].

This study also observed a general decline in femicide incidence rates over the ten-year period, from 16.1/100,000 in 2009 to 8.9/100,000 in 2018. This is in line with the findings of three national mortuary-based retrospective South African which reported a decrease trend in the femicide rate from 1999 (24.7/100 000) to 2017 (11.2 /100 000) [3, 14]. Molefe et al. showed similar trend results, with an average femicide incidence rate of 12.4/100,000 in her Cape Town based ten-year study (between 2000 and 2009), showed no significant annual differences [4]. Interestingly, this study showed an outlying increase in 2013 and 2016 was noted, which may warrant further investigation to understand the underlying causes.

Internationally, between 2010 and 2021, Europe witnessed an average reduction in femicide rates (by 19%), with the Americas recording an average increase over the same period (by 6%) [1]. Research in Africa between 2010 and 2019, unfortunately showed South Africa to consistently be the leading country of femicide rates on the continent [15].

Age is a key factor in analyzing the risk of femicide victims [16]. This study revealed a median age of femicide victims at 44 years, with the majority (67.3%) being between 18 and 39 years old. This aligns with Abrahams et al. (2022), who reported a stable mean age of femicide victims in South Africa at 37.7 years [14]. This also aligns with the findings of three national mortuary-based retrospective South African studies (2001) from data from 1999 to 2017, that showed the most pronounced femicide age ranged 30 to 44 years old [17]. This is also in keeping with a ten-year retrospective Cape Town based, South African study (2016), which showed that adult femicide victims had a median age of 41 years, with those aged between 18 and 39 years old being most affected; accounting for the majority (71%) of cases [4]. Interestingly, Wen-Li Fong et al. (2016) reports a majority femicide age incidence of 30-49-year-old in Taiwan [9], slightly older than this studies results.

Racial composition showed an overwhelming majority of victims being Black (97.2%), reflecting the demographic distribution of the region and possibly indicating socio-economic vulnerabilities. According to the 2011 South African Census, there were around 75.40% black, 20.08% white, 2.01% colored and 1.84% Indian people living within the borders of Tshwane [18]. This racial composition reflects both the demographics of the Tshwane region and the enduring legacies of apartheid, which structurally limited economic opportunities, access to education, healthcare, and other essential services for black South Africans. Despite progress since apartheid, these disparities persist, making black women and girls more susceptible to economic marginalization, social exclusion, and, consequently, heightened risk factors for gender-based violence.

When looking at the incidence regarding race distribution, a 1999 National femicide South African study showed that the femicide rate of a colored woman was double (18.3 / 100 000) than that of an African woman (8.9 / 100 000), and six times that of a white woman [4]. Molefe et al. further reported that black and colored femicide victims accounted for almost equal proportions of 46.9% and 45.7% of their Cape Town based study, respectively. The minority of victims in her study were white and Asian, in keeping with the demographical distribution of race in that study sample [4]. It is important to note, according to Campbell et al. (2003), that race is not independently linked to an increased risk of femicide rate, but that it is rather linked to socio-economic status [19].

When focusing on circumstances surrounding femicides, Molefe et al. showed that the highest number of cases occurred in July (9.9%), followed by November and December (similar percentages of 9.5%) in Cape Town. It furthermore showed that femicide numbers were similar across the months of February, April and June (percentages ranging between 7.6% and 7.7%) with the lowest being in January (5.6%) [4].

In investigating other demographic risk factors in South Africa, it is important to note that each municipal area is allocated the local South African Police Service (SAPS) station according to the Theoretical Human Resource Requirement (THRR). The SAPS stations are therefore an accurate predictor of the geographic locations, and for some areas can furthermore be an indirect predictor of the victims’ socio-economic profile [20]. A Cape Town based study showed that vast majority of femicide victims (30.8%) died in medical centers from 2006 to 2016. Furthermore, other common locations noted in the same Cape Town study showed victim’s homes (10.6%), victim’s formal housing (9.3%), urban public roads (8.3%) and 4.9% of victims were found in open land [4].

This study showed that the highest time of death was from 00h00 to 03h59 (63.2%), followed by 20h00 to 00h59 (15,7%). This similarly corresponds to a Cape Town based study, showing that over one third of femicide cases occurred in the late-night hours to early morning hours, with the most prevalent killing period being between 20h00 and 00h00 [4]. No other data on timing of femicides, both locally and internationally, was found at the time of this study.

Understanding the epidemiological and demographic variables in femicide victims is important, but what data is available regarding the pathology of these femicide victims? This study found gunshot wounds (GSWs) to be the most common cause of femicide, followed by blunt force trauma and sharp force injuries. This was consistent with the South African Firearms Control Briefing (2022), which reported an increase in firearm-related femicides from 17.3% in 2009 to 21.8% in 2017 [3, 14]. Similarly, in 2008, a 13-year retrospective South African study showed that the majority cause of death of femicide cases was also gunshot wounds (80%), followed by sharp objects (14%), and lastly blunt objects (11%) [21]. Alarmingly, firearms are also regarded as the most common weapons used in femicides in USA [8]. Furthermore, this study observed a relatively consistent linear trend in GSW’s over the study period, differing from Molefe et al. (2016), who reported a declining rate of gun-related femicide numbers between 2000 and 2009, by a total of 66.7% [4].

Gunshot-related femicides are common in South Africa due to the widespread availability of firearms and weak enforcement of gun control laws [22]. In contrast, countries with stricter firearm regulations, such as in Europe, see more femicides involving stabbing or strangulation, due to the stricter gun restrictions [23]. The ease of access to guns in South Africa makes them the weapon of choice in domestic violence, highlighting the need for stronger firearm regulations to reduce the amount of femicides. This study showed sharp force as the third highest manner of death, with a relatively stable trend over the 10-year period, with a sharp spike in 2013. Similarly, the South African follow up femicide study reported a steady trend in stab related femicide deaths between 1999 (32.7%) and 2009 (33.4%) [14]. Molefe et al. (between 2000 and 2009) showed a similar linear trend of stab injuries as causes of femicide death, during her ten-year Western-Cape study [4].

Previous femicide studies have also reported strangulation to the neck as a common form of interpersonal VAW, as it is a very personal region of the body [24]. Wen-Li Fong et al. found that strangulation /smothering, followed by sharp force injuries, to be the most frequent causes of death in a Taiwanese based study [9]. Violent asphyxias were notably found in 27.3% of femicides in a 2012 Egyptian study, mostly by throttling and strangulation alone or a combination of both [25]. This study showed that only 11.7% of femicides were strangulation related, being the fourth highest cause of death. A Western-Cape, South African study of femicides showed that a vast majority (35.7%) of victims died from asphyxial deaths, including strangulation [4].

Previous femicide studies also indicated that the location of the head and neck, being the most common injury site [24]. This study showed that the head, neck, and chest were the primary regions involved in fatal injuries, with GSWs predominantly affecting the head and chest. This pattern is supported by previous studies, such as the Taiwanese study by Wen-Li Fong et al., which also highlighted the neck and face as common sites of injury in femicides [9]. No other body location with manner of death data was found at the time of the study.

## Conclusion

This study demonstrates the high incidence and specific characteristics of femicides in the North-west area of Tshwane. The data results corroborate the introduction’s emphasis on the prevalence of firearm and sharp instrument use in these homicides and highlight significant geographic disparities in femicide rates. The consistent injury patterns identified important forensic insights and underscore the need for targeted public health and law enforcement interventions. By addressing these specific areas, it is possible to make strides in reducing the incidence of femicide and improving the safety and well-being of women in this region. This study proves useful, as government initiatives like the National Strategic Plan on Gender Based Violence and Femicide launched in 2020 by the Department of Women, Youth and Persons with Disabilities, are using a more evidence-based GBV prevention approach [26].

Future research should continue to monitor these trends and evaluate the effectiveness of implemented interventions. A coordinated approach involving law enforcement, healthcare providers, forensic experts, and social services is also crucial to comprehensively understand the distinction between intimate and non-intimate femicide, allowing for more effective interventions. Additionally, exploring the socio-economic and cultural factors contributing to femicide could offer deeper insights into prevention strategies. The findings of this study underscore the urgent need for comprehensive approaches to tackle the persistent and deadly issue of femicide in South Africa.

## Data Availability

Confidentiality of data information was maintained, as all post-mortem reports and files were kept in an access-controlled room during data selection and collection. The data collected and captured is stored on an access-controlled password protected device. Access to information about individual participants is restricted to the researcher only.
